# Guanine derivatives as promising candidates for the development of purine-based antimalarial drugs

**DOI:** 10.3389/fpara.2025.1634209

**Published:** 2025-07-30

**Authors:** Worlanyo Tashie, Harry P. de Koning, Nancy O. Duah-Quashie, Neils B. Quashie

**Affiliations:** ^1^ West African Centre for Cell Biology of Infectious Pathogens, Department of Biochemistry, Cell and Molecular Biology, College of Basic and Applied Sciences, University of Ghana, Accra, Ghana; ^2^ School of Infection and Immunity, College of Medical, Veterinary and Life Sciences, University of Glasgow, Glasgow, United Kingdom; ^3^ Department of Epidemiology, Noguchi Memorial Institute for Medical Research, University of Ghana, Accra, Ghana; ^4^ Centre for Tropical Clinical Pharmacology and Therapeutics, University of Ghana Medical School, Accra, Ghana

**Keywords:** *Plasmodium falciparum*, purine salvage system, purine analogues, *in vitro* sensitivity testing, PfENT1 protein, drug discovery, nucleotide metabolism, purine antimetabolites

## Abstract

**Introduction:**

The increasing resistance of *Plasmodium falciparum* to existing antimalarial drugs drives the urgent need for novel therapeutic strategies. The purine salvage pathway in *P. falciparum* is essential for the parasite’s survival due to its complete reliance on host-derived purines for nucleic acid synthesis and other essential processes. Although the purine salvage system has been intensively researched, no purine-based antimalarial drugs have been taken into preclinical development. The current study evaluated the chemotherapeutic potential of some purine nucleobase analogues against *P. falciparum*.

**Methods:**

*In vitro* sensitivity assays were conducted using the 72-hour SYBR Green drug assay on laboratory-adapted *P. falciparum* strains 3D7 and Dd2. The most potent nucleobase analogues were docked into PfENT1 using the PyRx software suite.

**Results:**

The analogues 8-azaguanine, 7-deazaguanine, and 6-thioguanine exhibited average EC_50_ values of 1.71 µM, 14.9 µM and 15.7 µM, respectively, for 3D7 and 5.2 µM, 16.3 µM and 18.6 µM, respectively, for the Dd2 strain, and subsequently tested against field isolates of *P. falciparum*. These *ex vivo* tests showed EC_50_ values ranging from 0.5 - 4.5 µM for 8-azaguanine, 3.8 - 12.3 µM for 7-deazaguanine, and 4.1 - 15.0 µM for 6-thioguanine. To understand their cellular targeting, molecular docking of the same analogues was performed using the structure of *P. falciparum* Equilibrative Nucleoside Transporter 1 (*Pf*ENT1). This demonstrated that guanine, 8-azaguanine and 7-deazaguanine formed five hydrogen bonds each with the same amino acid residues of *Pf*ENT1, whereas 6-thioguanine’s orientation allowed only two hydrogen bonds with *Pf*ENT1. The binding pose of inosine was different from these nucleobases.

**Discussion:**

These findings highlight the potential of guanine-based scaffolds, particularly 8-azaguanine and 7-deazaguanine, as promising leads for purine-based antimalarial drug development and the versatility of the *Pf*ENT1 transporter in the uptake of purine antimetabolites.

## 1 Introduction

Malaria remains a major global health challenge, with an estimated 263 million cases and 597,000 deaths reported in 2023 alone (WHO, 2024). Africa bears the highest burden, accounting for approximately 94% of all cases and 95% of deaths due to malaria. Children aged below 5 years accounted for 76% of the deaths due to malaria recorded from Africa in 2023. Among the five *Plasmodium* species that infect humans, *Plasmodium falciparum* is the most virulent and is responsible for the majority of malaria-related deaths (Basu and Sahi, 2017; Phillips et al., 2017; WHO, 2024).

While antimalarial resistance is nothing new, the recent emergence of artemisinin partial resistance, which first emerged in South-East Asia and currently in parts of Africa, is particularly alarming and threatens ongoing efforts in the control and elimination of malaria (Balikagala et al., 2021; Ward et al., 2022; Assefa et al., 2024; Rosenthal et al., 2024; WHO, 2024). This necessitates the urgent need for novel therapeutic strategies to tackle the rising threat of drug-resistant malaria (Rosenthal et al., 2024).

One promising strategy lies in targeting the parasite’s absolute dependency on host-derived purines for survival. *P. falciparum* cannot synthesize purines *de novo* and relies on salvaging them from host erythrocytes to support essential processes including DNA and RNA synthesis (Baldwin et al., 2007; Frame et al., 2015a). Purine import into the parasite, which is mediated by Equilibrative Nucleoside Transporters (*Pf*ENTs), is a critical step in the purine salvage pathway. *Pf*ENT1 serves as the primary route for purine uptake in *P. falciparum* and is essential for the parasite’s intraerythrocytic survival. This transporter has been validated as a promising target for antimalarial drug development (El Bissati et al., 2006; Quashie et al., 2008; Frame et al., 2015b; Zhang et al., 2018).

Purine analogues have demonstrated therapeutic efficacy in treating cancers, viral infections, and protozoan diseases (Muggia et al., 2012; Hulpia et al., 2019b; Pastuch-Gawołek et al., 2019; Natto et al., 2021; Huang et al., 2022; Fiuza et al., 2022). However, despite their broad utility, no purine-based antimalarial drugs have yet reached clinical use. Research has focused on nucleoside analogues including powerful transition state inhibitors of *P. falciparum* purine nucleoside phosphorylase (*Pf*PNP) such as Immuncillin-H, which displays picomolar K*_d_* for *Pf*PNP but a much lower IC_50_ for parasite growth (Ting et al., 2005), presumably because of poor uptake of such analogues by the parasite. We prioritized nucleobases over nucleosides due to their direct role in the parasite’s purine metabolism. Oxopurine nucleobases are activated via phosphoribosylation by hypoxanthine-guanine-xanthine-phosphoribosyl transferase (*Pf*HGXPRT), providing a 1-step incorporation into the nucleotide pool. In contrast, nucleosides, if they are to become nucleotides, must first be catabolized into their respective nucleobases by *Pf*PNPs, which introduces an additional and potentially rate-limiting step (Cassera et al., 2011; Ducati et al., 2013; Cheviet et al., 2019). This streamlined activation pathway makes nucleobases promising candidates for therapeutic intervention in *P. falciparum*. This study evaluated the chemotherapeutic potential of purine nucleobase analogues against *P. falciparum* as a proof of concept.

## 2 Materials and methods

### 2.1 Ethical consideration

The study was approved by the Institutional Review Board of the Noguchi Memorial Institute for Medical Research (NMIMR-IRB CPN: 045/23-24) and the Ethical Review Committee of the Ho Municipal Hospital in Ghana. Before enrolment, the study protocol was thoroughly explained to participants or their parents/guardians, and written informed consent was obtained from each participant and/or their guardian.

### 2.2 Parasite lines and field isolates

Cryopreserved *P. falciparum* 3D7 (chloroquine-sensitive) and Dd2 (chloroquine-resistant) laboratory-adapted strains as well as randomly collected *P. falciparum* clinical isolates were used. The laboratory-adapted strains were acquired from the Department of Epidemiology, Noguchi Memorial Institute for Medical Research, University of Ghana. The clinical isolates were collected from individuals who accessed services at the Municipal Hospital in Ho, Ghana. Participants were randomly recruited based on the detection of *P. falciparum* mono-infection by microscopy and presented with a parasite density between 1,000 and 250,000 parasites/µL of blood. Individuals on any antimalarial medication at the time of recruitment were excluded. Approximately 2 mL of venous blood was drawn from each participant into Acid Citrate Dextrose vacutainers and immediately transported to the laboratory for analysis.

### 2.3 Purine analogues

The purine nucleobase analogues evaluated in this study were obtained commercially. These include the following: 1-methylguanine (Fluka Chemika), 2,6-diaminopurine (Sigma-Aldrich), 2,6-dichloropurine (Aldrich Chemistry), 2,8-dimercapto-6-hydroxypurine (Avocado research Chemicals), 2-chlorohypoxanthine (Aldrich Chemistry), 2-hydroxypurine (Sigma-Aldrich), 6-amino-7-deazapurine (Aldrich Chemistry), 6-dimethylaminopurine (Aldrich Chemistry), 6-methoxypurine (Sigma-Aldrich), 6-thioguanine (Thermo Scientific), 7-deazaguanine (Sigma Aldrich), 7-deazahypoxanthine (Alfa Aesar), 8-azaadenine (Sigma-Aldrich), 8-azaguanine (Sigma Aldrich), 8-azahypoxanthine (Sigma-Aldrich), and 9-methylguanine (Fluka Chemika).

### 2.4 Culturing of *P. falciparum*

The *in vitro* continuous culture of *P. falciparum* (3D7 and Dd2 strains) was carried out using the Trager and Jensen (1976) method with slight modifications. Fresh, non-infected human erythrocytes were infected with *P. falciparum* parasites and maintained in complete Roswell Park Memorial Institute (RPMI) 1640 media under standard conditions. The culture system consisted of a 5% hematocrit suspension of purified O Rh “D” positive non-infected human erythrocytes in RPMI 1640 media supplemented with the following components: 6 g/L of HEPES (Sigma), 2 g of dextrose (Sigma), 50 mg/mL hypoxanthine (Sigma), 2 g/L of sodium carbonate (NaHCO_3_; Sigma), and 5 g/L of Albumax II (Sigma). Cultures were incubated at 37°C in an atmosphere of 1% O_2_, 3% CO_2_, and 96% N_2_. The medium was changed daily, and parasitemia levels were monitored through the microscopic examination of Giemsa-stained thin blood smears following protocols described by Moll and colleagues (Moll et al., 2013). Parasite cultures were maintained at parasitemia levels below 5%.

### 2.5 *In vitro* drug sensitivity assay

The sensitivities of *P. falciparum* 3D7 and Dd2 strains to purine nucleobase analogues were assessed using the 72-hour SYBR Green drug assay, as described by Moll and colleagues (Moll et al., 2013), with slight modifications.

#### 2.5.1 Compound preparation

Test compounds were initially dissolved in 100% dimethyl sulfoxide to yield stock concentrations of 20 mM. These stock solutions were diluted in incomplete RPMI 1640 medium to prepare working solutions with final in-well concentrations ranging from 0.39 µM to 100 µM.

#### 2.5.2 Plate set-up

Drug assays were conducted in 96-well opaque, flat-bottomed plates. For each compound, 200 μL of twice the maximum starting concentration (2 × 100 µM) was added to the second column (well) of a row. Serial two-fold dilutions were then carried out across the row from the 2^nd^ to the 10^th^ well to generate a concentration gradient. The 11^th^ well served as the negative control (no drug), while artesunate was used as a positive control.

#### 2.5.3 Parasite culture and inoculation

*P. falciparum* cultures were adjusted to 1% parasitemia and 1% hematocrit using human erythrocytes. A volume of 100 μL of this parasite suspension was added to each well, resulting in a final volume of 200 μL per well.

#### 2.5.4 Incubation

Plates were placed in a modular incubation chamber gassed with a mixture of 1% O_2_, 3% CO_2_, and 96% N_2_, and incubated at 37°C for 72 hours.

#### 2.5.5 Fluorescence measurement

After incubation, 100 μL of lysis buffer containing SYBR Green was added to each well. The plates were gently mixed, covered with aluminum foil, and incubated at room temperature in the dark for one hour. Fluorescence was measured using a FLUOstar Omega microplate reader (BMG Labtech, USA) with excitation and emission filters set at 485 nm and 520 nm, respectively.

#### 2.5.6 Data analysis

Fluorescence readings were exported to Microsoft Excel and analyzed in GraphPad Prism version 9.0 (GraphPad Software, USA). Dose-response curves were generated using a four-parameter logistic regression model (log[test compound] versus fluorescence) to determine EC_50_ values. Each assay was conducted in duplicate to ensure reproducibility.

### 2.6 *Ex vivo* drug sensitivity assay technique

The sensitivity of *P. falciparum* field isolates to purine nucleobase analogues was assessed using the 72-hour SYBR Green drug assay, essentially as described in Section 2.5. Field isolates were diluted 20-fold by mixing 1 part of the isolate with 19 parts of complete RPMI 1640 medium. The final-well concentrations of the test compounds ranged from 0.39 µM to 100 µM. All other steps, including the preparation of test compounds, plate set-up, incubation conditions, SYBR Green staining, fluorescence measurement and data analysis were identical to the *in vitro* assay protocol described in Section 2.5. Each compound was tested in duplicate to ensure reproducibility, and artesunate was included as an internal control.

### 2.7 Cytotoxicity assay

The cytotoxic effects of the potent purine analogues were evaluated on uninfected erythrocytes using a modified version of the 3-(4,5-dimethylthiazol-2-yl)-2,5-diphenyltetrazolium bromide (MTT)-based colorimetric assay, as described by Appiah-Opong and colleagues (Appiah-Opong et al., 2022; Appiah-Opong et al., 2011).

Briefly, 100 µL of each purine analogue, at concentrations ranging from 6.25 µM to 100 µM, was dispensed into separate wells of a 96-well microtiter plate. Subsequently, 100 µL of uninfected erythrocytes at 2% hematocrit was added to each well. Control wells containing 100 µL of each purine analogue in 100 µL of complete RPMI 1640 medium were set up in parallel for background signal correction.

The plates were incubated at 37°C for 72 hours in a modular incubation chamber gassed with a mixture of 1% O_2_, 3% CO_2_, and 96% N_2_. After incubation, 40 µL of 2.5 mg/mL MTT solution (prepared in phosphate-buffered saline) was added to each well in the dark. The plates were gently mixed and incubated for an additional 2 hours at 37°C.

At the end of the incubation period, absorbance was measured at 570 nm using the FLUOstar Omega microplate reader. All experiments were performed in triplicate. The 50% cytotoxic concentrations (CC_50_) of each compound were determined by non-linear regression analysis. Selectivity indices were calculated as the ratio of CC_50_ to EC_50_.

### 2.8 Molecular docking studies

The structure-activity relationship of potent purine nucleobase analogues identified through *in vitro* sensitivity testing was investigated using molecular docking. Docking studies were conducted with PyRx-v0.8, a virtual screening tool which has an in-built AutoDock-Vina for docking simulations (Dallakyan and Olson, 2015). Within PyRx, AutoDock Vina was used to compute binding affinities and generate docking poses of ligands in the defined binding pocket of *Pf*ENT1. The tool automates several steps, including file format conversion, energy minimization, and execution of the Vina engine with user-specified parameters.

#### 2.8.1 Preparation of protein target and ligands for docking studies

**Figure 1:**
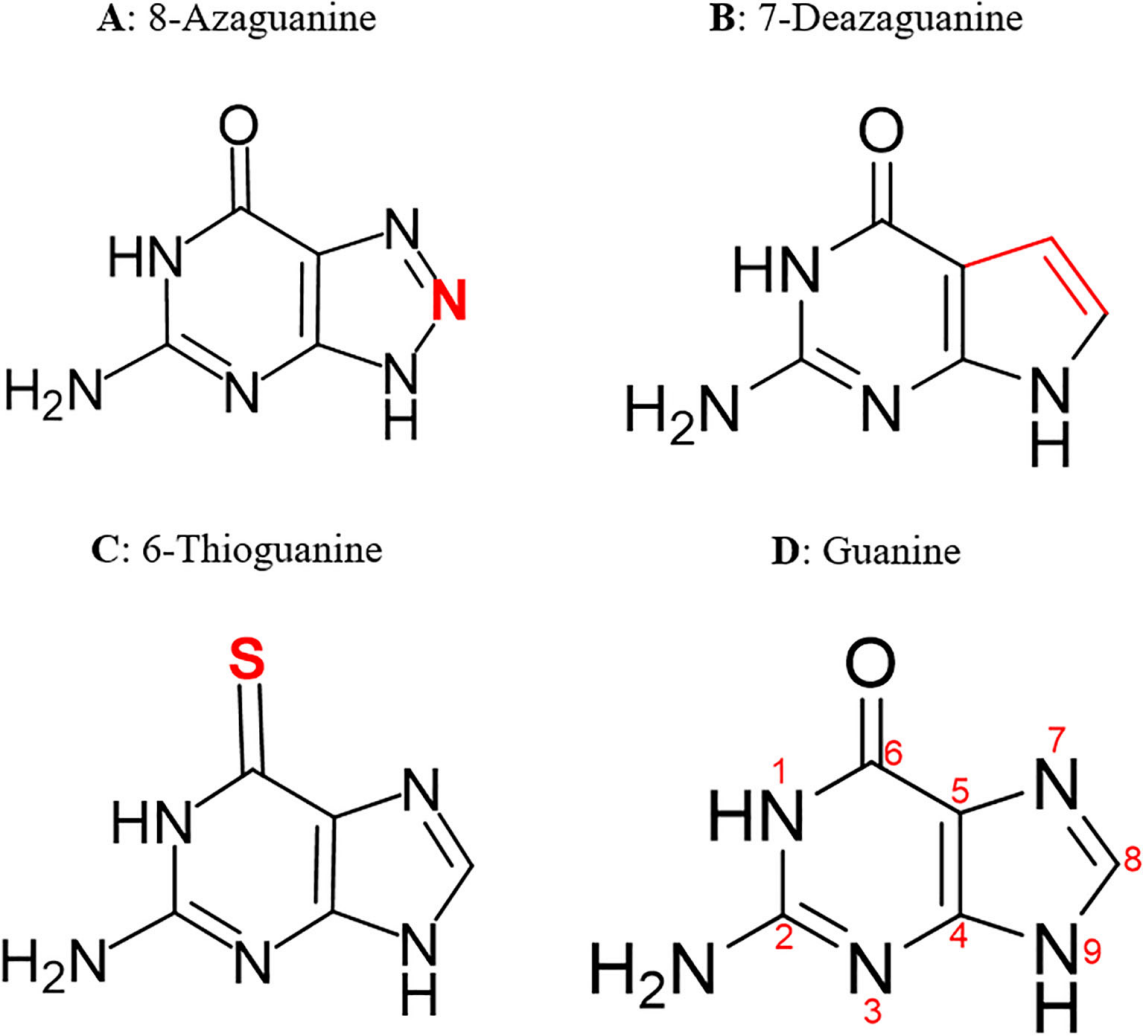
2D structures of the most potent guanine derivatives. Structural modifications to the guanine scaffold are highlighted in red.

The cryo-electron microscopy (cryo-EM)-resolved 3D structure of *Pf*ENT1 in complex with nanobody 48 and inosine (PDB code: 7WN1) (Wang et al., 2023) was retrieved from the RCSB-PDB database in PDB format. The chemical structures of the ligands were drawn using ChemDraw (version 23.1.1) and subsequently imported into Chem3D (version 23.1.1) for 3D optimization. The structures were then saved in Structure-Data File format. Preparation and refining of the *Pf*ENT1 protein, such as removal of native ligand and assigning hydrogen polarities and charges were performed using UCSF ChimeraX, adhering to the protocol described by Meng and colleagues (Meng et al., 2023). This was followed by energy minimization and geometry optimization of the proteins’ structures using PyRx-v0.8. Using the in-built Open Babel in PyRx, all the ligand structures were converted to Autodock suitable PDBQT format, and their energy minimized using Universal Forcefield (UFF). The 2D structures of the most potent guanine derivatives identified in this study are shown in **Figure 1**.

#### 2.8.2 Molecular docking of *Pf*ENT1 with guanine derivatives

Before proceeding with ligand docking, a redocking procedure was performed to validate the docking protocol by reproducing the original ligand poses observed in the cryo-EM structure. Each ligand was individually docked into the binding site of the prepared *Pf*ENT1 protein. The binding site was predicted using the COACH-D server (Wu et al., 2018), and a grid box was defined with the following parameters, chosen to be sufficiently large to include the entire protein-binding site of *Pf*ENT1: a center of 117.515×119.996×116.537 Å, and dimensions of 69.9708×48.6471×53.2414 Å. Docking was performed using AutoDock Vina within PyRx, with the “exhaustiveness” parameter set to 8 to improve search thoroughness. All other parameters were maintained at their default settings Docking results were clustered based on the root mean square deviation criterion. Complexes with a root mean square deviation of 0.0 were selected for further analysis. The best protein-ligand poses were visualized using Discovery Studio 2021 (BIOVIA). Key interactions within the active site were analyzed to identify significant contacts between the ligands and critical amino acid residues anchoring the compounds.

## 3 Results

### 3.1 *In vitro* sensitivity assay of 3D7 and Dd2 strains using purine analogues

All test compounds were initially screened *in vitro* using the 72-hour SYBR Green assay. Analogues that demonstrated inhibitory activity against *P. falciparum* were subsequently validated in biologically independent replicate experiments. The EC_50_ values obtained from these replicates exhibited minimal variation, with no statistically significant differences observed between experiments (*p* > 0.05).

**Figure 2:**
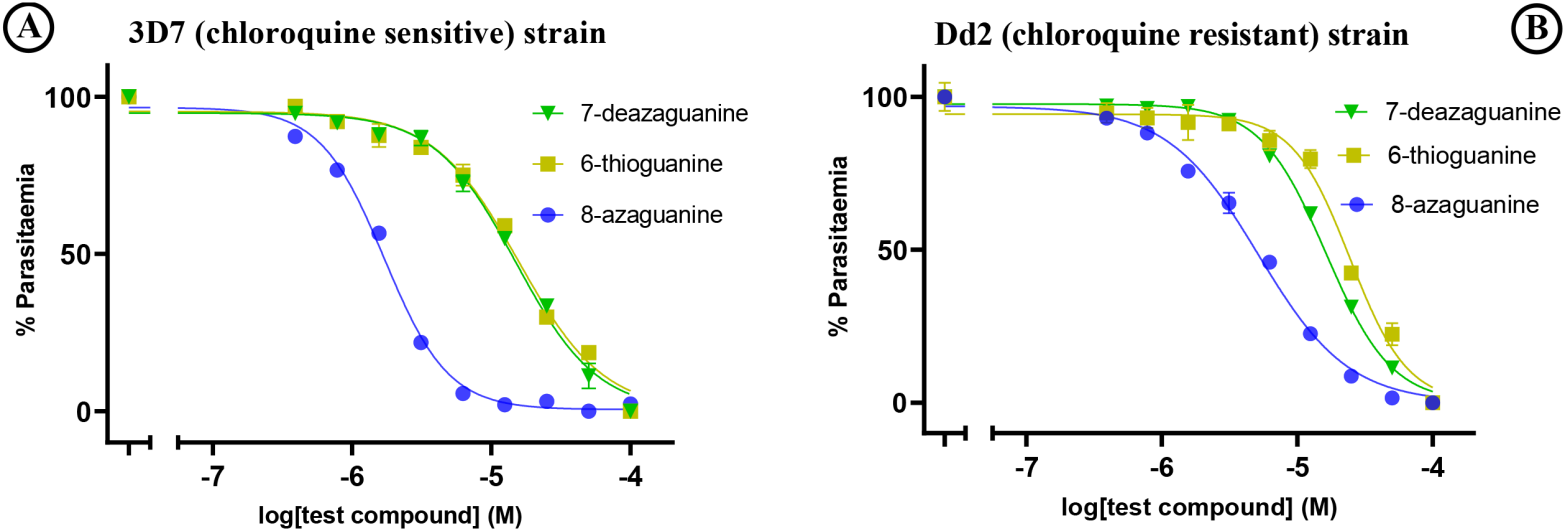
*In vitro* sensitivity testing of **(A)** 3D7 and **(B)** Dd2 strains against test purine nucleobase analogues. The graphs show a single representative experiment in duplicate; error bars are SEM.

Among the tested purine nucleobase analogues, only four - 8-azaguanine, 7-deazaguanine, 6-thioguanine, and 2,6-dichloropurine - demonstrated activity against the *in vitro* growth of *P. falciparum* parasites. Their EC_50_ values were 1.71 µM, 14.9 µM, 15.7 µM and 45.5 µM, respectively, for the 3D7 strain, and 5.2 µM, 16.3 µM, 18.6 µM and 36.19 µM, respectively, for the Dd2 strain. There was a borderline significant difference (*p* = 0.025) in the sensitivities of the 3D7 and Dd2 strains to 8-azaguanine but not to the other purine nucleobases (**Table 1**) and we conclude that purine derivates are not cross-resistant with chloroquine. The graphical representation of the EC_50_ of the potent guanine derivatives obtained from the *in vitro* experiments is shown in **Figure 2**.

**Table 1:** Effect of test purine nucleobases on 3D7 and Dd2 strains of *P. falciparum in vitro*.

Purine analogue	*In vitro* antiplasmodial activity EC_50_ values (µM)		Resistance factor	p-value
	3D7 strain	Dd2 strain		
8-azaguanine	1.71 ± 0.03	5.2 ± 0.6	3.04	0.025
7-deazaguanine	14.9 ± 0.6	16.3 ± 0.6	1.09	0.249
6-thioguanine	15.7 ± 0.1	18.6 ± 0.1	1.18	0.125
2,6-dichloropurine	45.5 ± 2.4	36.2 ± 4.8	0.79	0.221
Artesunate	0.006 ± 0.001	0.010 ± 0.001	1.77	0.001

All EC_50_ values are presented as averages in µM (mean ± SEM) of two independent measurements. An unpaired t-test was used to compare the *in vitro* sensitivities of the 3D7 strains and the Dd2 strains with NT3-KO. RF, Resistance factor. RF is calculated as a ratio of EC_50_ in resistant strain (Dd2) to sensitive strain (3D7).

### 3.2 *Ex vivo* testing of field isolates of *P. falciparum*

**Figure 3:**
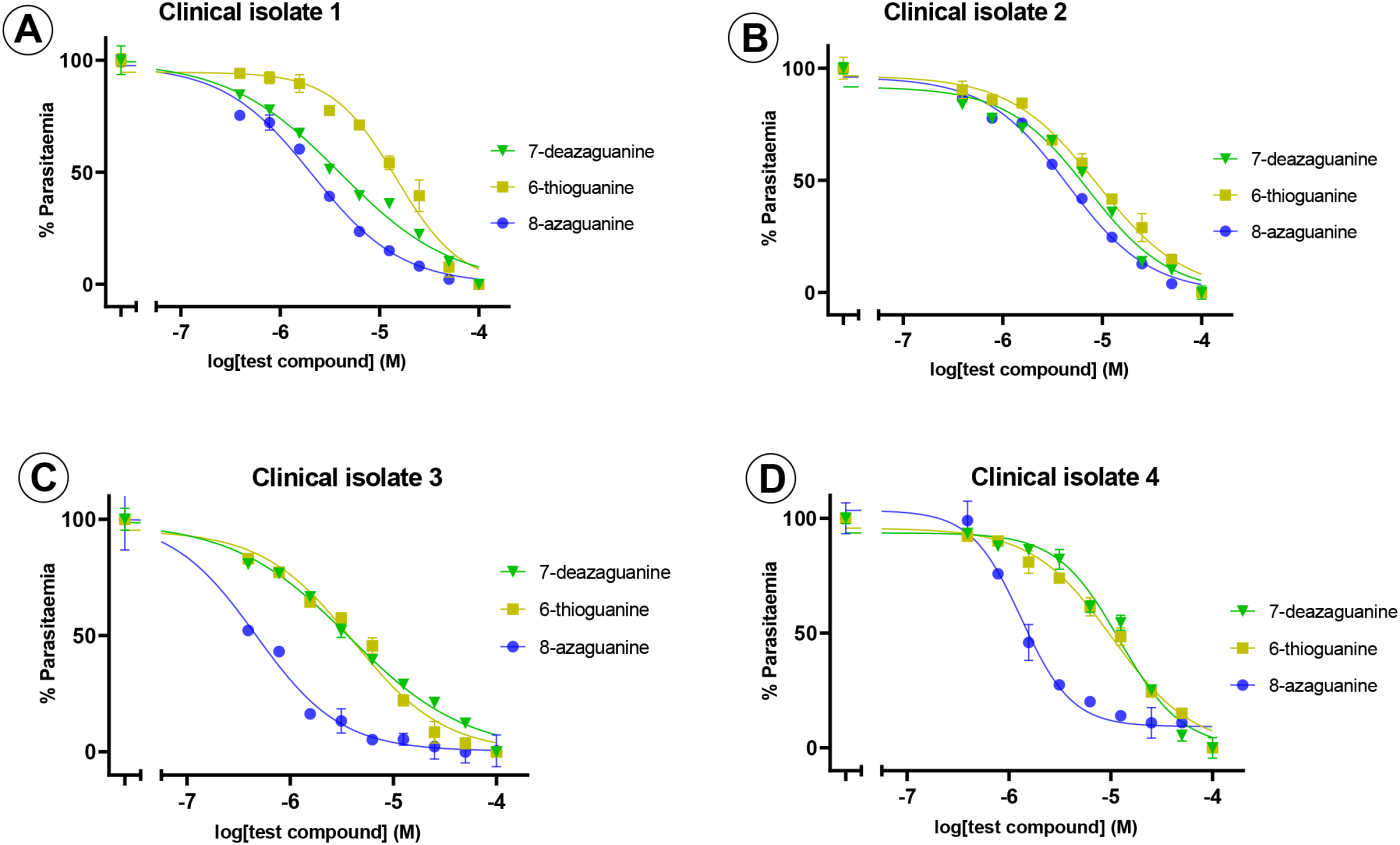
*Ex vivo* sensitivity testing of field isolates of *P. falciparum* against purine nucleobase analogues. The graphs show a single representative experiment in duplicate; error bars are SEM. Frames **(A–D)** show clinical isolates 1–4.

Following the initial screening of purine nucleobase analogues against 3D7 and Dd2 strains, only four compounds inhibited the *in vitro* growth of the parasites at low micromolar concentrations: 8-azaguanine, 6-thioguanine, 7-deazaguanine, and 2,6-dichloropurine. Notably, three of these active compounds shared a guanine scaffold and exhibited the most potent inhibitory effects. Therefore, based on these observations, the guanine derivatives were selected for further testing against four *P. falciparum* field isolates. 8-Azaguanine was the most potent analogue from the *ex vivo* testing, with EC_50_ values ranging from 0.5 - 4.5 µM across all isolates. This was followed by 7-deazaguanine, which had EC_50_ values ranging from 3.8 - 12.3 µM, whereas 6-thioguanine had a larger range of EC_50_ values on the field isolates, with EC_50_ values from 4.1 - 15.0 µM with a single isolate being slightly more sensitive to this analogue than to 7-deazaguanine. The *ex vivo* testing confirmed the efficacy of these three analogues against field isolates of *P. falciparum* and confirmed 8-azaguanine as the most potent of the analogues tested (**Figure 3**; **Table 2**).

**Table 2:** E*x vivo* results of the effect of purine nucleobases on *P. falciparum* field isolates.

Purine analogue	EC_50_ values (µM)				EC_50_ values range (µM)
	Isolate 1	Isolate 2	Isolate 3	Isolate 4	
8-azaguanine	2.1 ± 0.1	4.5 ± 0.5	0.5 ± 0.2	1.3 ± 0.1	0.5 – 4.5
7-deazaguanine	4.0 ± 0.7	7.2 ± 1.3	3.8 ± 0.6	12.3 ± 2.1	3.8 – 12.3
6-thioguanine	15.0 ± 1.9	9.1 ± 1.3	4.1 ± 0.1	10.3 ± 0.3	4.1 – 15.0
Artesunate	0.009 ± 0.001	0.013 ± 0.001	0.003 ± 0.001	0.008 ± 0.001	0.003-0.013

All EC_50_ values are presented as averages in µM (mean ± SEM) of two independent measurements.

### 3.3 Cytotoxicity and selectivity indices of potent purine analogues

All three potent guanine derivatives exhibited CC_50_ values above 50 µM on uninfected erythrocytes at the highest tested analogue concentration of 100 µM, suggesting acceptable safety profiles. 7-Deazaguanine demonstrated the lowest cytotoxicity, *i.e.* the highest CC_50_, 125.7 ± 2.7 µM (n=3). 8-Azaguanine showed the highest overall selectivity, with selectivity indices of 33.59 against the 3D7 strain and 11.05 against Dd2 strain, indicating strong antiplasmodial activity relative to its cytotoxicity. All three analogues demonstrated selectivity indices above the recommended threshold of 2 (Appiah-Opong et al., 2022) which indicates a favourable therapeutic potential (**Table 3**).

**Table 3:** Cytotoxicity and selectivity indices of potent purine analogues.

Purine analogue	CC_50_ values (µM)	Selectivity index	
		3D7 strain	Dd2 strain
8-azaguanine	57.44 ± 2.01	33.59	11.05
7-deazaguanine	125.7 ± 2.72	8.44	3.52
6-thioguanine	65.46 ± 2.25	4.17	9.85

All CC_50_ values are presented as averages in µM (mean ± SEM) of three independent measurements. Selectivity index (SI) was calculated as a ratio of CC_50_ to EC_50_. SI >2 indicates good selectivity of a therapeutic agent (Appiah-Opong et al., 2022).

### 3.4 Molecular docking studies

To understand the protein-ligand interactions, the potent guanine derivatives identified from the *in vitro* drug sensitivity testing were further docked into *Pf*ENT1 protein.

#### 3.4.1 Redocking to validate the docking procedure

Redocking was performed to confirm that the docking protocol could be used to accurately replicate the experimental poses of the ligand in the binding site, thereby validating the method for subsequent docking experiments with purine nucleobases. This process assessed whether the docking program could recreate the original cryo-EM resolved 3D structure poses of the ligands using PyRx.

**Figure 4:**
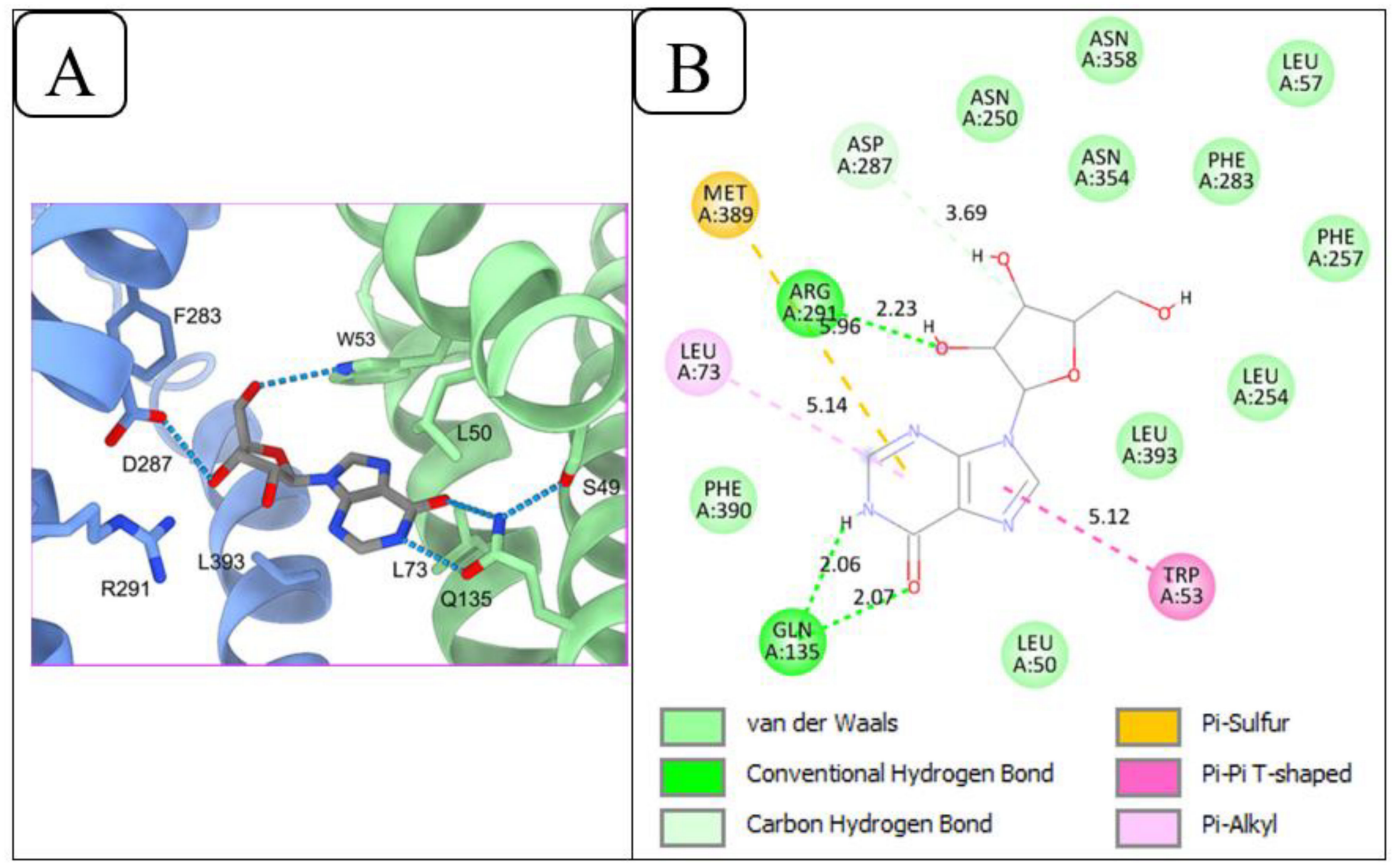
Redocking of *Pf*ENT1 and inosine to validate the docking procedure. **(A)** Inosine binding sites of *Pf*ENT1 from the cryo-EM solved 3D structure of the *Pf*ENT1-inosine complex, retrieved from the RCSB-PDB database. **(B)** Protein-ligand interactions of the redocked *Pf*ENT1 and inosine, performed using PyRx.

**Figure 4A** illustrates the protein-ligand interactions in the cryo-EM structure of the *Pf*ENT1-inosine complex, retrieved from the RCSB-PDB database (Wang et al., 2023). Wang and colleagues (Wang et al., 2023) reported that Gln135 of *Pf*ENT1 forms a hydrogen bond pair with the inosine keto group and N1(H), while Trp53 and Asp287, form hydrogen bonds with the 5’ and 3’ hydroxy groups, respectively, of the ribose moiety, and Arg291 further engages the 3’ and/or 2’ hydroxy moieties. Other residues observed by Wang and colleagues (Wang et al., 2023) to be involved in nucleoside recognition include Phe283 and Leu393. Additionally, the authors reported the following residues to be within 6 angstroms of the inosine’s ribose group: Asn250, Thr253, Asn354, and Asn358. The redocking procedure confirmed these interactions. As shown in **Figure 4B**, Gln135 of *Pf*ENT1 formed the same pair of hydrogen bonds with the 6-keto and N1(H) positions of inosine. Asp287 also engaged 3’-OH in the redocking, and Arg291 forms a hydrogen bond with 2’-OH. The only significant difference between the cryo-EM structure and the redocking is that in the latter Trp53 appears to engage in π-stacking with the purine ring rather than a hydrogen bond with the 5’-OH of ribose; this difference depends on the orientation of this tryptophan’s indole ring system within the binding pocket, which in turn would depend on whether the energy gain from the 5’-OH hydrogen bond or the π-π interaction is stronger.

These findings show that the redocking process yields outputs close to the experimentally observed poses, validating the docking procedure for further protein-ligand interaction studies.

#### 3.4.2 Molecular docking results of the guanine scaffolds

**Figure 5:**
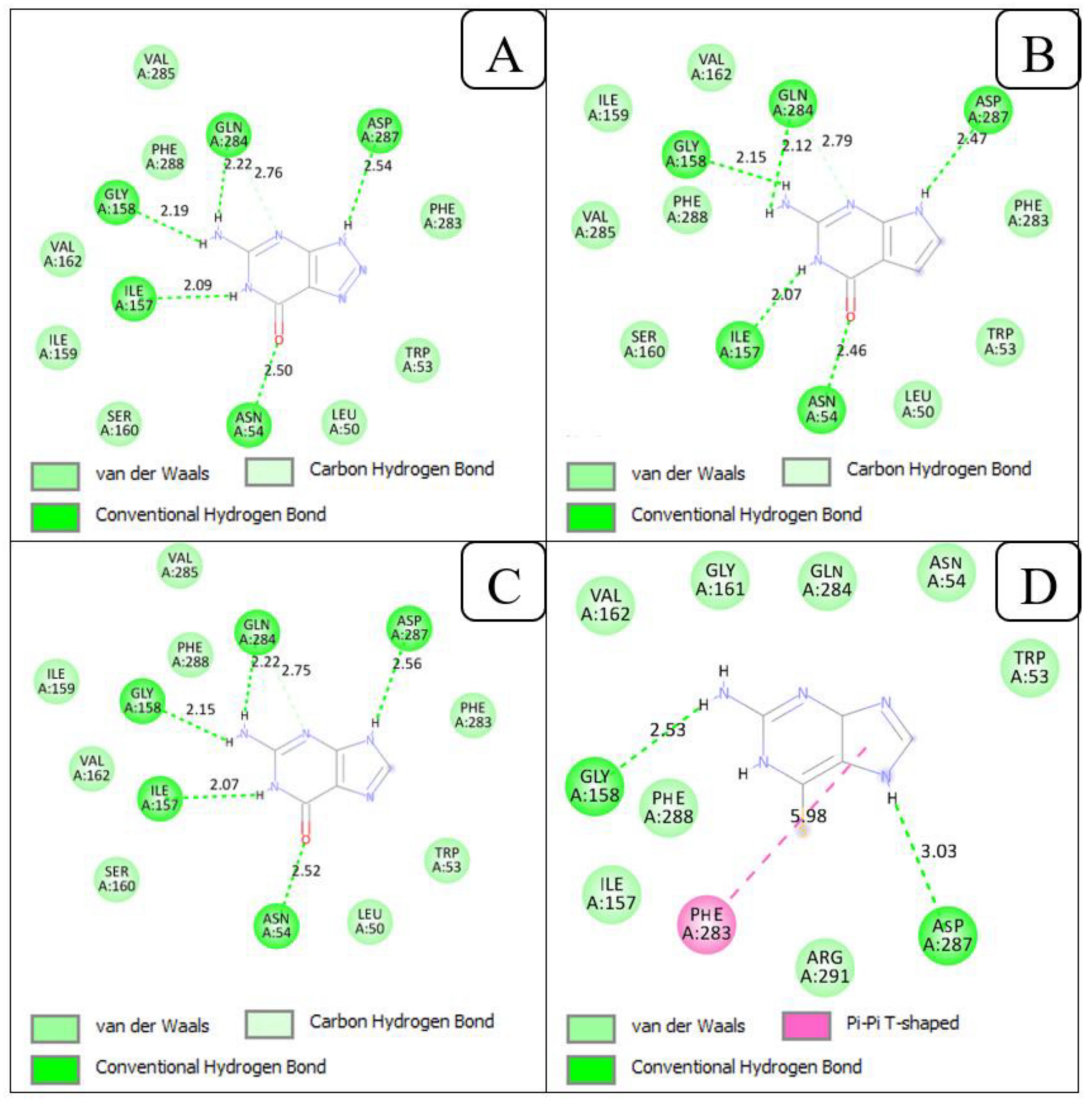
2D view of the interactions of *Pf*ENT1 protein with the guanine scaffolds. **(A)** Protein-ligand interactions of *Pf*ENT1 and 8-azaguanine. **(B)** Protein-ligand interactions of *Pf*ENT1 and 7-deazaguanine. **(C)** Protein-ligand interactions of *Pf*ENT1 and guanine. **(D)** Protein-ligand interactions of *Pf*ENT1 and 6-thioguanine.

The interactions of 8-azaguanine and 7-deazaguanine with *Pf*ENT1 were similar. *Pf*ENT1 formed five (Rosenthal et al., 2024) hydrogen bonds with both 8-azaguanine and 7-deazaguanine via the following amino acid residues: Asn54, Ile157, Gly158, Gln284 and Asp287 (**Figures 5A, B**). Both ligands further made Van der Waals contact with *Pf*ENT1 through the amino acid residues Leu50, Trp53, Ile159, Ser160, Val162, Phe283, Gln285, and Phe288. Docking guanine into *Pf*ENT1 yielded protein-ligand interactions identical to the binding of 8-azaguanine and 7-deazaguanine (**Figure 5C**). Thus, the two most potent nucleobase analogues interacted identically with *Pf*ENT1, yet in a very different orientation from inosine, illustrated perhaps most clearly by the fact that Asp287 interacts with 3’-OH of inosine but with N(9)H in the guanine analogues, and Gln135, the only residue contributing H-bonds with the oxopurine base moiety in inosine (to 6-keto and N(1)H), is not involved at all in binding the guanine analogues. Instead the 6-keto group now forms a hydrogen bond with Asn54 and N(1)H with Ile157 – neither being involved in the binding of inosine. Interestingly, the 2-amine moiety of the guanine analogues, which does not exist in inosine, contributes two strong H-bonds, with Gly158 and Gln284, which also are not engaged in inosine binding. Clearly, the two oxopurine nucleobase analogues interact with *Pf*ENT1 very differently from the oxopurine nucleoside, with scarce overlap between the binding positions.

The orientation of 6-thioguanine has shifted again (**Figure 5D**), highlighting the importance of the Asn54 H-bond with the 6-keto oxygen for the positioning of guanine nucleobases in this transporter, as the thio moiety does not form strong hydrogen bonds (Civcir, 2001). The positional shift is further enabled by the dominant tautomeric form of 6-thioguanine including the protonation of N7 rather than N9 as is the case for 7-deazaguanine and 8-azaguanine (Contreras and Madariaga, 1998; Maciejczyk and Pyrka, 2023), resulting in the strong Asp287 H-bond with N (7)H in 6-thioguanine rather than N (Baldwin et al., 2007)H. The change in position results in the further loss of the H-bonds by Ile157 (to N(1)H) and Gln284 to the 2-amine but the binding pose is stabilized by a new π-π-hydrophobic interaction with the Phe283 side chain, just as adenine is stabilized by Phe aromatic interactions in the binding pocket of the *Trypanosoma brucei* TbAT1/P2 transporter (Munday et al., 2015), and Gly158 maintains H-bond with the 2-amine. The contribution of the 2-amine moiety in the binding of guanine and its analogues is consistent with our previous observation of approximately 3-fold higher affinity by *Pf*ENT1 for guanine than to hypoxanthine, just like the bonding by five strong H-bonds (distance between 2.56 and 2.07 Å) is consistent with the extraordinarily low K*_i_* value for guanine of 0.11 ± 0.01 µM (Quashie et al., 2008), corresponding to a Gibbs free energy of interaction (ΔG^0^) of −39.7 kJ/mol. In addition to the hydrogen bonds, the amino acid residues, Trp53, Asn54, Ile157, Gly161, Val162, Gln284, Phe288 and Arg291 increased the strength of the interaction between *Pf*ENT1 and 6-thioguanine through Van der Waals interactions.

## 4 Discussion

Purine analogues have been utilized in clinical settings for the treatment of cancers and viral infections (Muggia et al., 2012; Jordheim et al., 2013; Pastuch-Gawołek et al., 2019), and are under development against certain protozoan diseases (Hulpia et al., 2019b; Fiuza et al., 2022). Despite significant efforts to target the purine transport system in the development of antimalarial drugs (Quashie et al., 2010; Frame et al., 2015a; Zhang et al., 2018; Sosa et al., 2019), no purine-based antimalarial drugs are currently available in clinical practice. Here, we evaluated the chemotherapeutic potential of some purine nucleobase analogues against *P. falciparum* isolates and use them to explore the issues of purine drug uptake in *Plasmodium*.

Our findings revealed that guanine-derived analogues, specifically 8-azaguanine, 7-deazaguanine, and 6-thioguanine, inhibited the *in vitro* growth of *P. falciparum* at low micromolar concentrations. The potent activity of these guanine derivatives against *P. falciparum* is likely mediated through one of several mechanisms. These may include inhibition of *Pf*ENTs, or disruption of key enzymes involved in purine salvage metabolism within the parasite, or these compounds may act as “subversive” substrates for the parasite’s purine salvage enzymes, resulting in incorporation into DNA or RNA (Galmarini et al., 2002; Pruijssers and Denison, 2019; Rehan et al., 2019; Thomson and Lamont, 2019; Borbone et al., 2021; Kamzeeva et al., 2023). This pathway would commence with the conversion to monophosphate nucleotides by *Pf*HGXPRT followed by further enzymatic steps to the analogues of (2’-deoxy)ATP and/or (2’-deoxy)GTP (Cassera et al., 2011; Ducati et al., 2013; Cheviet et al., 2019), which would eventually impair nucleic acid synthesis and/or structure and ultimately lead to parasite death.

The analogue 8-azaguanine was the most potent analogue identified in this study. 8-Azaguanine is an azapurine, and previous studies have demonstrated that azapurines are important analogues with a broad spectrum of anti-infective and antineoplastic activity (Hahn, 1979; Giorgi and Scartoni, 2009; Wang et al., 2016; Hou et al., 2020), acting by incorporation into RNA (Nelson et al., 1975). For instance, 8-azapurines possess a broad spectrum of biological activity and are studied for their interactions with many enzymes and receptors for their antitumour and antiviral activity (Giorgi and Scartoni, 2009; Wang et al., 2016; Kim et al., 2019). Despite the observed potency of 8-azaguanine and 6-thioguanine against *P. falciparum* parasites, a possible challenge with their direct usage as antimalarial drugs is their reported toxicities to human cells when used in cancer treatments (Berman et al., 1985; Wang et al., 2016; Kim et al., 2019; Tseligka et al., 2023). 8-Azaguanine and 6-thioguanine are approved drugs that have been previously used in the treatment of acute leukaemias (Colsky et al., 1955; Wang et al., 2016; Kim et al., 2019). However, both analogues are toxic to human cells, posing a challenge to their continuous usage in cancer treatment (Berman et al., 1985; Wang et al., 2016; Kim et al., 2019; Tseligka et al., 2023). 8-Azaguanine produces toxicity to human cells through its incorporation into RNA, whereas 6-thioguanine produces toxicity to human cells through its incorporation into DNA (Keough et al., 2006; Kim et al., 2019). It is, however, worth noting that the short-term administration of these potent guanine analogues for severe malaria cases is less likely to cause the toxicities associated with extended use, as seen with prolonged cancer treatment regimens. Moreover, an effective antimalarial dosage may stay well below what is required in cancer chemotherapy, especially when used in combination with another antimalarial agent. Thus, these potent guanine analogues against *P. falciparum* infection remain promising, especially as the starting point for further drug discovery efforts.

Cytotoxicity testing of the analogues on human erythrocytes indicated acceptable safety profiles, with 8-azaguanine exhibiting the highest selectivity and 7-deazaguanine demonstrating the lowest cytotoxicity. Thus, with a favourable toxicity profile, the 7-deazaguanine scaffold could become a preferred candidate for the development of purine-based antimalarial drugs just like 7-substituted, 7-deazapurine nucleosides have provided excellent scaffolds against cancer and viral infections (Perlíková and Hocek, 2017), and against both human and animal forms of African and American trypanosomiasis (Hulpia et al., 2019a; Hulpia et al., 2020; Fiuza et al., 2022; Mabille et al., 2022). In contrast to these other cell types, *P. falciparum* only incorporates nucleobases into their nucleotide pool, through *Pf*HGXPRT, with nucleosides first degraded to oxopurine nucleobases (Cassera et al., 2011), hence the decision to focus on the nucleobase moieties in this study. However, given that the 7-deazanucleosides are also believed to be active in their nucleotide form, for instance through phosphorylation by adenosine kinase in kinetoplastid protozoa or mammalian cells (Perlíková and Hocek, 2017; Hulpia et al., 2019b), the nucleoside and and nucleobase prodrugs ultimately converge to the same active compound *in cellulo*.

Further exploration of the structure-activity relationships of the antimalarial activity of both compounds by medicinal chemistry, aided by mechanism of action and structural docking studies, should be able to improve the antimalarial activities of these potent guanine analogues and potentially reduce human cell toxicity: note, for instance, that 7-deazaadenosine (tubercidin) is toxic to human cells but most 7-substituted tubercidin analogues (Hulpia et al., 2019a) and 7-deazainosine derivatives (Hulpia et al., 2020) are not. Tubercidin and the 7-substituted tubercidin sangivamycine also display potent antiplasmodial activity but are not selective (Coomber et al., 1994). Thus, a more promising starting point for medicinal chemistry exploration may be to combine the active modifications to guanine, for instance, 6-thio,8-azaguanine, 6-thio,7-deazaguanine and 7-deaza,8-azaguanine, like combining the tubercidin and cordycepin scaffolds yielded 3’-deoxy,7-deazaadenosine, a highly potent trypanocide that is curative *in vivo* with low dose oral administration, even in models of cerebral sleeping sickness (Hulpia et al., 2019b). Importantly, it has already been established that 8-azaguanine and 6-thioguanine are very good substrates of *Pf*HGXPRT, which shows 336 and 19.6-fold higher catalytic efficiency (k_cat_/K_m_), respectively, than the human HGPRT for these analogues, although it has >5-fold *lower* catalytic efficiency for guanine itself (Keough et al., 2006). This clearly shows that *Pf*HGXPRT, performing the critical phosphoribosylation step from guanine (analogue) to GMP (analogue), can easily accommodate changes at oxopurine nucleobase position 6, and the 8-aza modification. Moreover, 7-deaza,8-azaguanine is the guanine analogue of allopurinol (7-deaza,8-azahypoxanthine), which is in use as an antileishmanial agent and not toxic to humans – it is widely used as treatment for gout – because it is not incorporated into the nucleotide pool by human purine metabolic enzymes (Day et al., 2007), but is incorporated into RNA in *Leishmania* and *Trypanosoma* species (Looker et al., 1986). Allopurinol is also an excellent substrates for protozoan nucleoside transporters (Al-Salabi et al., 2003; Natto et al., 2005). In *Plasmodium*, a combination with allopurinol improved the efficacy of quinine in acute complicated malaria (Sarma et al., 1998).

The limited amino acid sequence similarity between the *Pf*ENTs and hENTs (Arora et al., 2016) further provides the possibility of optimizing these potent analogues to improve their efficacy and selectivity for *Pf*ENTs over the hENTs. More important than the sequence dissimilarity, ENT transporters have been widely documented to have highly different and exclusive substrate selectivities even within the same organism. For example, in *Leishmania* species, the NT1.1 and NT1.2 transporters are selective for adenosine and uridine, whereas NT2 recognises only the oxopurine nucleosides inosine and guanosine (Aldfer et al., 2022), despite 32% identity/52% similarity on amino acid sequence. Indeed, a single amino acid replacement can alter the substrate or inhibitor preferences of a transport protein as shown for instance for the UapA nucleobase transporter of *Aspergillus nidulans* (Koukaki et al., 2005) and for the *T. brucei* pentamidine transporter TbAQP2 (Alghamdi et al., 2020).

To gain further insight into the interaction modes of the potent guanine derivatives with *Pf*ENTs, molecular docking studies were carried out. The docking of a cryo-EM-resolved 3D structure of *Pf*ENT1 (Wang et al., 2023) with the potent guanine derivatives identified in this study revealed that guanine, 8-azaguanine, 7-deazaguanine all formed five hydrogen bonds with the same amino acid residues of *Pf*ENT1. Hydrogen bonds, due to their specificity, are critical in biomolecular interactions and contribute significantly to the stability and selectivity of target-ligand complexes (Murali et al., 2023). The binding mode of guanine by *Pf*ENT1, then, appears to rely primarily on H-bonds with the 6-keto group, the 2-amine moiety and N1(H), i.e. the pyrimidine half of the purine ring, in addition to the H-bond between Asp287 and N (Baldwin et al., 2007)H. The fact that this is identical to the reported binding mode of the facilitative nucleobase transporter hFNT1 present in the human erythrocyte membrane (Wallace et al., 2002), apart from the N (Baldwin et al., 2007)H interaction, is a significant advantage in the design of effectively targeted guanine analogues against intra-erythrocytic *P. falciparum*. It contrasts the binding mode of the nucleobase transporters of the kinetoplastid species *T. brucei* (TbH2 (Wallace et al., 2002),, *T. cruzi* (TcrNT2 (Aldfer et al., 2024), and *Leishmania major* (LmajNBT1 (Al-Salabi et al., 2003),, all of which form very strong hydrogen bonds with N7.

The protein-ligand interactions of *Pf*ENT1 with these guanine derivatives highlighted some specific positions on the guanine ring that can be exploited in designing purine-based antimalarial drugs that are specific and selective for the parasite. With positions 1, 2, 6 and 9 in 8-azaguanine and 7-deazaguanine identified as essential for high affinity towards *Pf*ENT1, positions 3, 7, and 8 offer flexibility for potential structural optimization. These findings align with the observations of Wang and colleagues (Wang et al., 2023), who demonstrated that positions 1 and 6 of inosine’s nucleobase moiety are critical for binding, while positions 3, 7, and 8 exhibit flexibility for further optimization. For 6-thioguanine, which assumes a different orientation in the *Pf*ENT1 binding pocket, positions 1, 3, 6 and 9 are potentially available for modifications. Interestingly, inosine, guanine and 6-thioguanine each interact with a distinct set of amino acid residues in the binding pocket, showing an extraordinary versatility of substrate for an ENT-family transporter. Our data are completely consistent with the reports by multiple research groups that this carrier can accommodate both nucleosides and nucleobases, and oxopurines as well as the aminopurine nucleoside adenosine (Carter et al., 2000; Parker et al., 2000; Quashie et al., 2008). The structural studies presented here and by Wang and colleagues (Wang et al., 2023) provide the clearest rationale to date for these observations. For instance, the glutamine residue binding inosine with a pair of H-bonds with 6-keto and N1(H) would equally be able to bond with the 6-amine/N1 motif of adenosine. And although inosine binding involves H-bonds to the ribose hydroxy moieties, which would be expected to correlate with low affinity for nucleobases, this is compensated for by a shift of the purine ring position allowing the formation of several new H-bonds. The single substitution of the guanine keto group with a thio moiety initiated yet another orientation for energy optimization. All this demonstrates that *Pf*ENT1 is quintessentially suited as a conduit or target for purine antimetabolites, consistent with the observations of Frame and colleagues (Frame et al., 2015b).

While recent reports show that 7-deaza-7-substituted nucleosides possess extraordinarily potent and selective and antiprotozoal activity (Hulpia et al., 2019a; Hulpia et al., 2020; Natto et al., 2021; Fiuza et al., 2022; Lin et al., 2022), the antimalarial effects and/or selectivity of these nucleosides has been disappointing (Serge Van Calenbergh and Guy Caljon, unpublished). However, we stress that this is most likely because, uniquely in *Plasmodium*, nucleosides must first be broken down by *Pf*PNPs before being phosphoribosylated into the nucleoside pool as nucleotides (Cheviet et al., 2019). This adds the necessity to accommodate the structure-activity relationships of additional enzymes to the compound design, and the different structure-activity relationships may be mutually exclusive. Thus, our results would justify an exploration of the antimalarial activity of 7-deaza,7-substituted oxopurine nucleobases for antimalarial activity similar to those of the corresponding nucleosides against many other protozoa.

In conclusion, the guanine derivatives 7-deazaguanine, 6-thioguanine, and particularly 8-azaguanine demonstrated significant inhibitory activity against the *in vitro* growth of *P. falciparum* at low micromolar concentrations. Our findings suggest that these guanine derivatives hold promise as potential starting points for the development of purine-based antimalarial drugs. Employing medicinal chemistry approaches to optimize these analogues could enhance their antimalarial efficacy and their selectivity for *Pf*ENT1. Further studies are necessary to elucidate the mechanisms through which these potent guanine derivatives and/or their metabolites inhibit *P. falciparum* growth. In addition, site-directed mutagenesis of *Pf*ENT1 could be used to validate the predicted binding interactions identified in this study and provide deeper insights into the structure-activity relationships of these analogues.

## Data Availability

The original contributions presented in the study are included in the article/supplementary material. Further inquiries can be directed to the corresponding author.
